# Supplementary material for: Multi‐dataset identification of innovative feature genes and molecular mechanisms in keratoconus
Source: J Cell Mol Med. 2024 Sep 19;28(18):e70079. doi: 10.1111/jcmm.70079 (PMC11412914; doi:10.1111/jcmm.70079)
Supplement: Supplementary file 2 — Material S2. [file JCMM-28-e70079-s002.zip › 7.GSEA analysis/3.DBNDD1/KEGG_AMINOACYL_TRNA_BIOSYNTHESIS.html]

Details for gene set KEGG\_AMINOACYL\_TRNA\_BIOSYNTHESIS[GSEA]

|  || Dataset | DBNDD1.group.cls#h\_versus\_l |
| Phenotype | group.cls#h\_versus\_l |
| Upregulated in class | h |
| GeneSet | KEGG\_AMINOACYL\_TRNA\_BIOSYNTHESIS |
| Enrichment Score (ES) | 0.21932168 |
| Normalized Enrichment Score (NES) | 0.65646166 |
| Nominal p-value | 0.92038834 |
| FDR q-value | 1.0 |
| FWER p-Value | 1.0 |
Table: GSEA Results Summary

  

Fig 1: Enrichment plot: KEGG\_AMINOACYL\_TRNA\_BIOSYNTHESIS      
 Profile of the Running ES Score & Positions of GeneSet Members on the Rank Ordered List

  

| SYMBOL | TITLE | RANK IN GENE LIST | RANK METRIC SCORE | RUNNING ES | CORE ENRICHMENT || 1 | SEPSECS | na | 6122 | 0.147 | -0.0303 | Yes |
| 2 | WARS2 | na | 9282 | 0.081 | -0.0406 | Yes |
| 3 | EARS2 | na | 9963 | 0.072 | 0.0111 | Yes |
| 4 | PARS2 | na | 10194 | 0.069 | 0.0727 | Yes |
| 5 | MTFMT | na | 13096 | 0.037 | 0.0263 | Yes |
| 6 | PSTK | na | 13585 | 0.033 | 0.0453 | Yes |
| 7 | CARS2 | na | 13848 | 0.031 | 0.0687 | Yes |
| 8 | LARS2 | na | 14280 | 0.028 | 0.0840 | Yes |
| 9 | IARS2 | na | 14930 | 0.023 | 0.0884 | Yes |
| 10 | NARS2 | na | 16930 | 0.010 | 0.0405 | Yes |
| 11 | DARS2 | na | 19174 | -0.006 | -0.0183 | Yes |
| 12 | FARSA | na | 19374 | -0.008 | -0.0165 | Yes |
| 13 | RARS2 | na | 19841 | -0.012 | -0.0185 | Yes |
| 14 | TARS2 | na | 21061 | -0.023 | -0.0311 | Yes |
| 15 | FARS2 | na | 21077 | -0.023 | -0.0090 | Yes |
| 16 | AARS2 | na | 21508 | -0.027 | 0.0054 | Yes |
| 17 | HARS2 | na | 22559 | -0.038 | 0.0127 | Yes |
| 18 | YARS2 | na | 22648 | -0.039 | 0.0488 | Yes |
| 19 | SARS2 | na | 22796 | -0.041 | 0.0850 | Yes |
| 20 | FARSB | na | 23234 | -0.046 | 0.1181 | Yes |
| 21 | MARS2 | na | 26846 | -0.104 | 0.1175 | Yes |
| 22 | VARS2 | na | 27116 | -0.110 | 0.2193 | Yes |
Table: GSEA details [plain text format]

  

Fig 2: KEGG\_AMINOACYL\_TRNA\_BIOSYNTHESIS      
 Blue-Pink O' Gram in the Space of the Analyzed GeneSet

  

Fig 3: KEGG\_AMINOACYL\_TRNA\_BIOSYNTHESIS: Random ES distribution      
 Gene set null distribution of ES for **KEGG\_AMINOACYL\_TRNA\_BIOSYNTHESIS**
